# Recent advances in gene-editing approaches for tackling antibiotic resistance threats: a review

**DOI:** 10.3389/fcimb.2024.1410115

**Published:** 2024-06-26

**Authors:** Amani H. Al-Fadhli, Wafaa Yousef Jamal

**Affiliations:** ^1^ Laboratory Sciences, Department of Medical, Faculty of Allied Health Sciences, Health Sciences Center (HSC), Kuwait University, Jabriya, Kuwait; ^2^ Department of Microbiology, College of Medicine, Kuwait University, Jabriya, Kuwait

**Keywords:** antibiotic resistance, genes, pathogens, infections, challenge

## Abstract

Antibiotic resistance, a known global health challenge, involves the flow of bacteria and their genes among animals, humans, and their surrounding environment. It occurs when bacteria evolve and become less responsive to the drugs designated to kill them, making infections harder to treat. Despite several obstacles preventing the spread of genes and bacteria, pathogens regularly acquire novel resistance factors from other species, which reduces their ability to prevent and treat such bacterial infections. This issue requires coordinated efforts in healthcare, research, and public awareness to address its impact on human health worldwide. This review outlines how recent advances in gene editing technology, especially CRISPR/Cas9, unveil a breakthrough in combating antibiotic resistance. Our focus will remain on the relationship between CRISPR/cas9 and its impact on antibiotic resistance and its related infections. Moreover, the prospects of this new advanced research and the challenges of adopting these technologies against infections will be outlined by exploring its different derivatives and discussing their advantages and limitations over others, thereby providing a corresponding reference for the control and prevention of the spread of antibiotic resistance.

## Introduction

The development of innovative gene editing and targeting strategies to combat bacterial infections has been a top priority for researchers ever since the first antibiotics were discovered. The tendency of pathogenic bacteria to develop antibiotic resistance has accelerated, and as a result, the world has had to put in place significant barriers to address this problem. Antibiotic-resistant bacteria were classified into 12 groups by the World Health Organization (WHO) in 2017. This categorization was based on their capacity to fend against infection. Among these, P. aeruginosa, A. baumannii, and Enterobacter spp. have been recognized as the most important pathogens that were shown to oversee high death rates in hospitals (Tacconelli et al., 2018). Two things that led to the emergence of such resistant bacteria were the misuse and abuse of antibiotics (Reygaert, 2018). Additional factors included the mechanisms by which bacteria develop resistance against antimicrobial drugs; examples of such mechanisms include random gene mutation (Davies and Davies, 2010), horizontal gene transfer (HGT) (Sun et al., 2019) as a means of transmitting resistance genes, changing the permeability or efflux of the drug (Murray et al., 2015), and the capacity to form biofilm (Lewis, 2007). Because persister cells are present, communities of bacteria responsible for long-term infections may develop an antibiotic tolerance, which could lead to chronic infections without changing the genetic composition of the bacterium. It is vital to create novel methods for identifying, managing, and preventing superbug infections due to the complex ways in which they spread. Numerous scientific investigations have highlighted the detrimental characteristics of these multidrug-resistant bacteria and several approaches to address them (Fischbach, 2011; Buckner et al., 2018; Annunziato, 2019).

As of right now, a few therapeutic strategies have been employed to address the resistance mechanisms displayed by antibiotic-resistant bacteria. These strategies include developing next-generation antibiotics, employment of efflux pump inhibitors (EPIs) (Ghosh et al., 2019; Mulani et al., 2019; Gray and Wenzel, 2020)/quorum sensing inhibitors (QSIs) as well as the manufacture of host defense peptides, which is a promising substitute for conventional antibiotics. But all these traditional methods have drawbacks (Alaoui Mdarhri et al., 2022), and a suitable solution to this issue has yet to be created (Suh et al., 2022).

## Confronting antibiotic resistance: contemporary strategies and solutions

Antibacterial resistance or Multidrug resistance (MDR) is the capacity of bacteria, when they become resistant to antibiotics where they should be killed (Ahmad et al., 2017, 2023). These types of bacteria are becoming more prevalent daily, but at the same time, the understanding of biology and technology is expanding. To survive under these different antibiotic settings, bacteria use intrinsic or acquired mechanisms to get different resistance genes (Blair et al., 2015). One of such resistance mechanisms is an expression or generation of resistance genes against different multiple targets, that significantly alter different growth conditions (Whittle et al., 2021). Some good examples that bring out DNA alteration or mutations in bacteria are mobile genetic elements (MGEs), phages or plasmids (Schroeder et al., 2018). As mentioned earlier also, MDR is divided into two main categories i.e. genetic and phenotypic (Allemailem, 2024). The mutations or alterations that occur in the bacterial DNA give rise to genetic resistance. Moreover, this type of bacterial resistance is also seen when the resistance genes exchange or enter between bacteria. On the other hand, the phenotypic MDR causes alterations within the bacteria but doesn’t bring out any change in its genetic makeup and usually disappears within the individual bacterial cell (Balaban et al., 2019; Allemailem, 2024).

A full understanding or compression of the molecular foundations of antibiotic resistance or MDRs is very important as it will help in the discovery of innovative therapies or novel treatments for antibiotic-resistant infectious diseases (Darby et al., 2023). Recent years have seen major advancements in the science behind how antibiotics work and how bacteria develop inhibitory resistance to their deadly and lethal effects (Ahmad et al., 2018; Ahmad et al., 2023). The three pillars of interrelated tactics that bacteria adopt to counteract antibiotics are tolerance, resistance, and persistence (Allemailem, 2024). The understanding of how the biochemical actions of different drugs or pharmacological compounds like antibiotics work and how bacteria pose resistance to them is still fully unknown but has advanced significantly. Numerous studies have explained various acts of resistance mechanisms that include the production of antibiotic resistance-related genes, downregulation or alteration of porins to reduce the entry or penetration of antibiotics through the bacterial cell, modification of its cell wall components or target sites, the inactivation of antibiotics and the hyperactivity of active efflux pumps (Alav et al., 2021; Morgan et al., 2021; Allemailem, 2024). Based on these above-mentioned mechanisms, several techniques have been proposed that include phage therapy (Lin et al., 2017; Kortright et al., 2019), drug-loaded nanoparticles (Rai et al., 2012; Makabenta et al., 2021), photodynamic therapy, combinatorial therapy, drug repurposing ( Rangel-Vega et al., 2015; Liu et al., 2021), antimicrobial peptides (Hassan et al., 2012; Lima et al., 2021) or anti-virulence compounds (Ménard et al., 2014).

Combinatorial therapy is the use of various pharmacological combinations as opposed to a single medication to target multiple areas and achieve a synergistic impact that kills the bacteria (Tamma et al., 2012). Combinatorial therapy is required due to the rapid emergence of antibiotic-resistant bacterial strains, as monotherapy is no longer effective in treating most bacterial infections, especially resistant ones. However, this technique impacts the pharmacokinetics and pharmacodynamics of the employed medications due to some incompatibility difficulties between distinct drugs (Petrosillo et al., 2010; León-Buitimea et al., 2020).

Apart from this, gene-blocking oligonucleotides and RNA interference (RNAi) are the two examples of RNA-based treatments used against AMR organisms. These methods take advantage of the enzymatic targeting of bacterial mRNA by oligonucleotides, which permits the removal of the genes that are responsible for resistance (Kole et al., 2012). Methods based on antisense RNA also seem to have provided a way to monitor the genes involved in growth promotion and MDR. However, there are certain toxicity problems and low intracellular absorption with RNA-based therapies (Kole et al., 2012). In certain cases, phage therapy has also shown promise in combating germs resistant to antibiotics. Nevertheless, there are several difficulties, such as how they interact with intracellular bacteria, encourage the formation of neutralizing antibodies, and cause bacteria to become resistant to phages (Doss et al., 2017). Monoclonal antibodies (mAbs) have also been used to treat some bacterial resistance but some of the barriers preventing their usage are bacterial target selection and degradation through bacterial proteolytic enzymes (Pelfrene et al., 2019).

The employment of gene editing tools, such as transcription activator-like effector nucleases (TALENs) and zinc finger nucleases (ZFNs), which may be utilized to precisely modify drug-resistant bacteria’s DNA, has also contributed to the resolution of antibiotic resistance problems. For their removal or cleavage, TALENs and ZFNs are employed against the target DNA sequences (Zhang et al., 2019; Li et al., 2020). The ZFN and TALEN tools have opened a new avenue for contemporary gene editing tactics. Some significant obstacles, including delivery difficulties, off-target consequences, and complexity, have prevented these genome-editing techniques from being widely successful.

Since its discovery, the CRISPR/Cas9 (Clustered Regularly Interspaced Short Palindromic Repeats-Cas9) gene editing system, which is currently regarded as the most inventive method, has been applied quickly to the treatment of antibiotic resistance or MDR. Since it is the quickest, least expensive, and most effective method of genome editing, it is widely employed. Additionally, it is employed in the improvement of genetic flaws, the eradication of major infectious viruses (Kim and Lee, 2022; Redman et al., 2016), and the removal of bacterial infections (Kang et al., 2017; Park et al., 2017). Numerous scientific researches support the adoption of CRISPR/Cas-based strategies to prevent the spread of MDR (Uribe et al., 2021; Wu et al., 2021; Kim & Lee, 2022).

## Unveiling CRISPR/Cas: a cutting-edge gene editing marvel and its intriguing contrasts with alternative technologies

Initially found in both archaea and bacteria, CRISPR-Cas was found to play a role in adaptive immunity (Ishino et al., 2018). Bacteria and archaea can use this mechanism to identify and eliminate viruses and plasmids that are entering their cells. CRISPR-Cas has been altered for use in gene editing and genetic engineering within the last ten years. CRISPR-Cas gene editing and base editing are two crucial methods for precisely modifying an organism’s DNA. These technological developments have made it possible to correct genetic defects, increase crop yields, and create cutting-edge biotech applications (Arora and Narula, 2017; Yao et al., 2018; Wu et al., 2020). Scientists can now conduct research and tests more efficiently because of technological improvements, which speed up the production of results. In this review, we will focus mainly on recent breakthroughs of CRISPR/cas9 and its derived gene editing technologies like CRISPRi, CRISPRa and CRISPR base editing in the context of antibiotic resistance. We will uncover the limitations and advantages of CRSIPR-based gene editing technology over other gene editing technologies and will conclude with some new thoughts and ideas that could help in making these technologies more efficient and precise to combat the tendency of bacterial antibiotic resistance.

Talking about other gene editing/targeting approaches, CRISPR/Cas-based techniques have become a widely adopted, promising complement against conventional approaches to fighting against antibiotic resistance with minimal harmful repercussions on humans or the environment. CRISPR-Cas genome editing was praised for its simplicity, effectiveness, adaptability, and lack of requirement for any markers for recognizing species of harmful bacteria as compared to other DNA-based genetic engineering techniques (Rabaan et al., 2023). Despite this, it can accurately target a particular sequence using just one guide RNA (gRNA) and the protein that accompanies it (Cas). This system usually acts as an adaptive immunity, shielding the body from genetic material that is not native to the body (Tetsch, 2017). However, its method of action is different for different resistant bacteria, which use it as a transferable or integrative system for attacking antibiotic-resistant genes (Wu et al., 2021).

Since it makes it possible to precisely and successfully identify the genes causing bacterial drug resistance, the development of CRISPR-based editing of genomes has had a profound effect on medicine. Previous research showed that a novel gene that renders bacteria extremely resistant to the last-resort group of antibiotics was found and driven out from *Escherichia coli* utilizing the type II CRISPR-Cas9 system (Sun et al., 2017). Moreover, other studies have also reported the use of Six csm and cas6 genes in a CRISPR-Cas-III-A system to target interference with crRNA processing in Mycobacterium tuberculosis (Wei et al., 2019). The CRISPR-Cas-III-A system was essentially created to cleave co-transcriptionally active DNA, and target and identify RNA to create a potent defense (Liu et al., 2018). Furthermore, the use of different CRISPR and its related strategies, like CRISPR-Cas-II-A in Streptococcus agalactiae (Lier et al., 2015) or a CRISPR-Cas12a strategy in conjunction with enzymes to identify genes resistant to kanamycin, ampicillin, and chloramphenicol, has confirmed genetic editing in a range of antibiotic-resistant bacteria (Chen et al., 2023). Conventional CRISPR-Cas-mediated gene editing is widely used, but it has some limitations. These include the potential for genomic instability due to inaccurate off-target and on-target editing, which can be caused by the low GC content of sgRNA, the use of protospacer adjacent motif (PAM-in) orientation, or the use of inefficient delivery methods to remove AMR genes (Javaid and Choi, 2021; Kundar and Gokarn, 2022).

**Figure 1:**
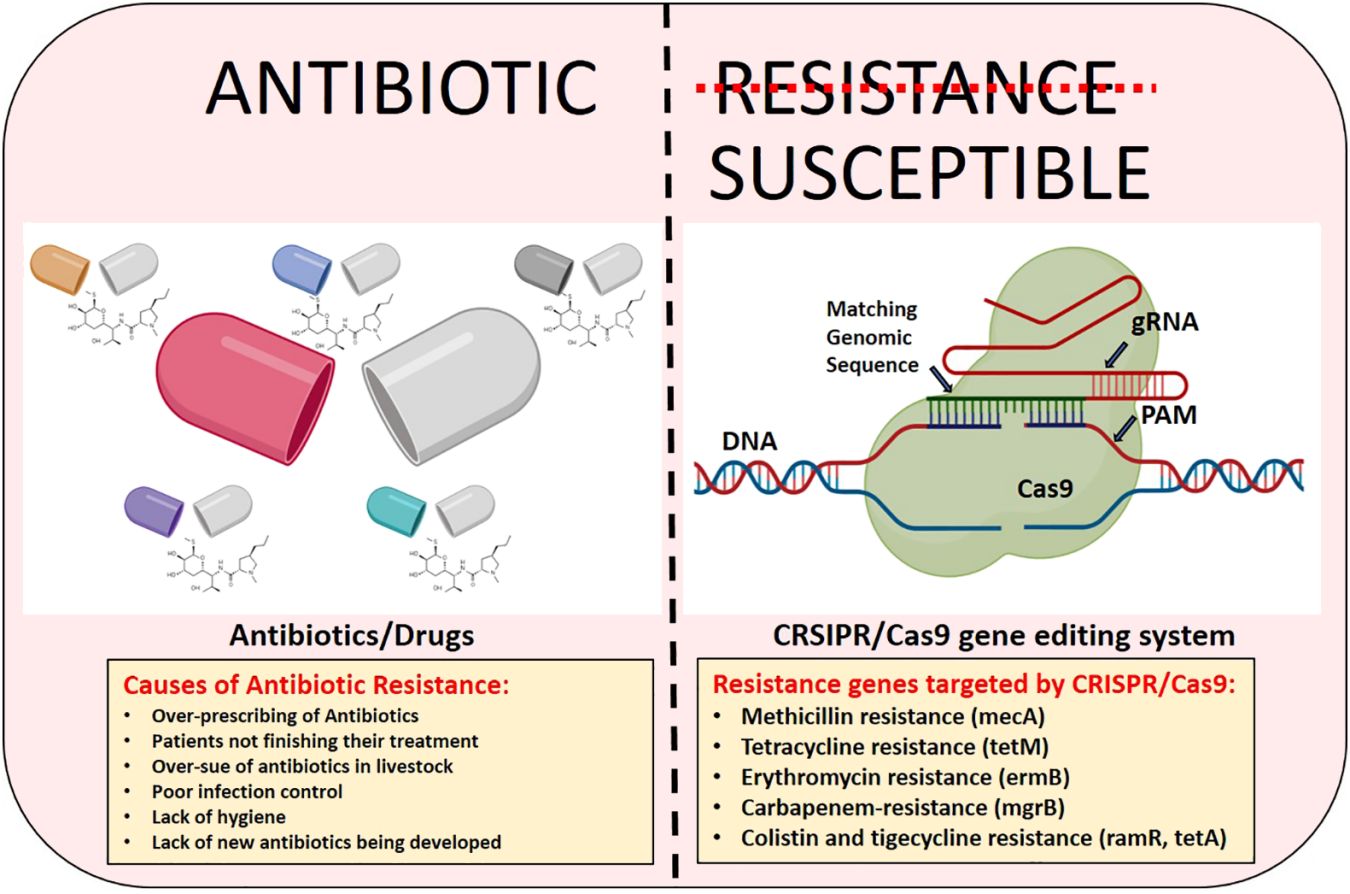
The visual depiction illustrating the causes of antibiotic resistance and the effectiveness of CRISPR/Cas9 gene editing technology in addressing it.

Base-editing techniques are currently being employed to address this problem by lowering the frequency of faults. With this technique, we can alter DNA sequences in a variety of ways without risking dsDNA cleavage. A modifying enzyme for precise nucleotide modification and an ssDNA for programmable DNA binding are the two main parts of base editors, that allow this method to change bases properly. DNA base editors use fusion proteins (nickase Cas9, dead Cas9, or dead Cas12a/b) fused to ssDNA-specific nucleobase deaminases to increase the efficacy of site-directed mutagenesis (Kantor et al., 2020; Rabaan et al., 2023). Moreover, it is very important to understand that the two gene editing methods that preceded the CRISPR/Cas 9 technology were TALEN and ZFN respectively. However, these two technologies have their different drawbacks as compared to CRISPR/Cas9. In the case of TALEN and ZFN, the main limitation lies in their designing that should be uniquely created to target each DNA target with the custom proteins. Furthermore, this method is also time-consuming, costly and labor-intensive as compared to CRISPR/Cas9 which impedes researchers or scientists from using them. While off-targeting is the limitation that comes with all gene editing technology, the additional complexities and intricacies of protein-DNA binding make ZFNs and TALENs even more susceptible to off-target cleavage. In the case of CRISPR/cas9 while off-target effects are still possible significant progress has been made in lowering off-target cleavage. Scientists and researchers are busy improving the specificity-enhancing alterations and better guide RNA designing to address this problem. **Figure 1** is the visual representation, depicting the causes of antibiotic resistance and the role of CRISPR/Cas9 in combating it.

Furthermore, CRISPR-Cas is also known for its great versatility, which can be achieved by effortless retargeting by modifying its guide RNA sequence, hence facilitating efficient multiplexing and concurrent editing of several different genes. On the other hand, ZFNs and TALENs are less adaptable and might need a substantial amount of reengineering for every new target as they are not as flexible as CRISPR/Cas9. Because of these qualities, CRISPR-Cas is a comparatively more dependable, durable, robust, and low-cost gene editing technique. CRISPRi (CRISPR interference or inhibition) and CRISPRa (CRISPR activation) are also considered the two derivatives of CRISPR/cas9 technology but they are more likely called as the handy tools that could manipulate the gene function by activating it or by inhibiting it. Some researchers have also shown the lead roles of CRISPRi in combating biofilm-based antibiotic-resistant bacterial infections. They tried to knock down the various genes that are either related to bacterial virulence or with their mechanism of action that could lead to those biofilm infections. The key genes they addressed were luxS (Zuberi et al., 2017b), fimH (Zuberi et al., 2017a) and bolA (Azam et al., 2020). They further showed the role of the OmpR/EnvZ pathway in controlling such infections with the help of this technology (Zuberi et al., 2022). Their work demonstrates the significant merit worthy of commendation in the context of this technology to combat such infections.

## The revolutionary progress of CRISPR/Cas9 against antibiotic resistance

Genome editing based on CRISPR/Cas9 is currently widely regarded as a potentially next-generation method to tackle infectious diseases, particularly the ones brought on by antibiotic-resistant bacteria (Bikard and Barrangou, 2017; Duan et al., 2021). CRISPR/Cas9 mediated genome editing may be applied as a gene-based strategy as well as a pathogen-targeted strategy, depending on the target gene’s location. The pathogen-based strategy depends on focusing on a few bacterial chromosomal regions. By using this technique, certain pathogenic strains are killed, and bacterial cells are destroyed. Targeting the antibiotic resistance genes harbored by different plasmids, however, is how the gene-focused approach is continued. This strategy makes the bacterium more susceptible to antibiotics (Allemailem, 2024). Pathogen-focused strategies can be utilized to treat certain strains of interest in heterogeneous bacterial pathogens in addition to specific infections; gene-focused strategies, on the other hand, are unclear. Nonetheless, this method pertains to the decrease in the prevalence of antibiotic resistance within the microbial community as a means of treating bacterial illnesses (Shabbir et al., 2019).

Unlike traditional antimicrobials, the CRISPR/Cas9 technology uses precise and very specific sequence targeting to distinguish between harmful and symbiotic organisms as already mentioned earlier. This method has shown success in transforming antibiotic-resistant strains of bacteria like Staphylococcus aureus and *Escherichia coli* with the help of a plasmid that encodes Cas9-driven RNA (Shabbir et al., 2019). Using this strategy, the expression of the antibiotic resistance gene was accurately reduced. However, since the goal of this strategy is to reach MDR treatment, it is now in the preclinical stage. Nevertheless, this genome-editing technology has also been used in several clinical investigations as an antibacterial medicine. Certain clinical isolates of S. aureus harboring gene resistant to methicillin (mecA) when treated with Cas9 and modified crRNA exhibited a roughly 50% reduction in sickness (Aslam et al., 2020; Palacios Araya et al., 2021). Parallel to this, a different study showed this gene editing method also targets the gene responsible for erythromycin resistance (ermB) that eventually reduces the growth of intestinal *E. faecalis* resistant to erythromycin. Moreover, using a mouse skin colonization model, CRISPR/Cas9 interventions dramatically decreased S. aureus skin colonization (Wang et al., 2019). One of the bacteria from the ESKAPE group of pathogens i.e. *Klebsiella pneumonia* can acquire several MDR mutations by natural horizontal gene transfer (Sun et al., 2019; Allemailem, 2024). In one research these bacteria were subjected to CRISPR/Cas9 mediated genome editing to investigate the roles of certain genes like ramR, tetA, and mgrB, which promote colistin and tigecycline within carbapenem-resistant K. pneumonia (Sun et al., 2019). These genes were rendered inactive because of the upregulation of CRISPR/Cas9, which changed the bacteria’s susceptibility to tigecycline and colistin, respectively.

Multi-drug resistant *E. faecalis* is devoid of the whole functional CRISPR system, particularly the Cas9 system (Palmer and Gilmore, 2010). Using the pheromone-responsive plasmid (PRP), the entire functional CRISPR/Cas9 system was successfully delivered to these multidrug-resistant bacteria. This method, which was exclusive to *E. faecalis*, produced effective conjugation. pD1, which is specific for the enterococcal antibiotic resistance genes like tetM (encoding tetracycline resistance) with ermB (encoding erythromycin resistance), was used to build a constitutively transcribed CRISPR/Cas9 system. *In vitro*, the erythromycin and tetracycline resistance of the E. *faecalis* bacterium was effectively reduced (Rodrigues et al., 2019). Concurrently, another *in vivo* intestinal colonization experiment demonstrated that PRP targeting ermB in donors may have decreased the incidence of intestinal *E. faecalis* that is resistant to erythromycin, supporting the use of engineered PRP in reducing multidrug resistance. The Gram-positive bacteria Enterococcus faecium is increasingly linked to antibiotic resistance in hospital-acquired infections. Higher recombination levels in these microbes with the help of CRISPR/Cas9 mediated DNA editing resulted in their specifically designed mutant clinical strain named E745 (de Maat et al., 2019).

Another study showed that TP114, a conjugative plasmid harboring a CRISPR/Cas9 system was improved for transfer efficiency and this method eliminated over 99.9% of the specific antibiotic-resistant *E. coli* in the gut microbiome of mice with a single dosage. The Citrobacter rodentium infection model was likewise treated with this approach, and after a few days of treatment, the infection was completely eradicated (Neil et al., 2021). Lysostaphin is a potent staphylolytic enzyme that can harm Staphylococcus aureus bacteria. This glycylglycine endopeptidase exhibits strong antibacterial properties. S. aureus does, however, exhibit a certain degree of resistance against this endopeptidase because of its wall teichoic acid (Wanner et al., 2017). Using CRISPR/dCas9, some researchers were able to suppress the transcription of the tarO, tarG, and tarH genes, prevent the synthesis of teichoic acid within the walls of bacteria, and kill S. aureus by sensitizing the bacterium to lysostaphin (Wu et al., 2019). In a unique study, blaKPC, blaNDM, and blaOXA-48 in carbapenem-resistant Enterobacteriaceae (CRE) were precisely cut using a CRISPR/Cas9-based plasmid-curing system (pCasCure). The outcomes showed that, with curative effectiveness of greater than 94%, pCasCure effectively cut these genes found in several Enterobacteriaceae species of clinical isolates of *E. coli, K. pneumonia, E. hormaechei, E. xiangfangensis, and S. marcescens* (Hao et al., 2020; Allemailem, 2024). Furthermore, the pKpQIL plasmid’s parA, repA, and repB genes were carefully removed to stop the plasmid-based carbapenemase resistance gene from desensitizing the impact of the carbapenem antibiotic on CRE. The MIC value was lowered by over eight times because of this experiment (Hao et al., 2020; Allemailem, 2024).

## Conclusion

In this review, we tried to explain the latest developments in gene editing technology, especially CRISPR/Cas9, which holds great promise against antibiotic resistance. We shed light to clarify how CRISPR and Cas9 interact to address the problem of antibiotic resistance, we examined the possibilities of these novel approaches in combating antibiotic resistance, highlighting their benefits and drawbacks in comparison to traditional tactics. Through providing an extensive synopsis of these developments together with an analysis of their obstacles, this review seeks to provide insightful information to direct initiatives to manage and stop the emergence of antibiotic resistance.

## Author contributions

AA-F: Writing – review & editing, Writing – original draft, Methodology, Formal analysis, Conceptualization. WJ: Writing – review & editing, Writing – original draft, Investigation, Formal analysis.
